# Supplementary material for: Fitness of the Papaya Mealybug, Paracoccus marginatus (Hemiptera: Pseudococcidae), after Transferring from Solanum tuberosum to Carica papaya, Ipomoea batatas, and Alternanthera philoxeroides
Source: Insects. 2022 Sep 2;13(9):804. doi: 10.3390/insects13090804 (PMC9505760; doi:10.3390/insects13090804)
Supplement: Supplementary file 1 [file insects-13-00804-s001.zip › insects-1856046- supplementary/Supplementary Material -Huiyu Chuai.pdf]

Supplementary Table 1. Developmental time of *Paracoccus marginatus* reared on four different host plants (F_0 - F_2).

Stage (d)	Generation	*Carica papaya*		*Ipomoea batatas*		*Solanum tuberosum*		*Alternanthera philoxeroides*	
		n	Mean ± SE	*n*	Mean ± SE	*n*	Mean ± SE	*n*	Mean ± SE
1st instar	F_0	88	5.50 ± 0.09dA	82	7.13 ± 0.18bA	91	5.84 ± 0.12cA	15	8.27 ± 0.28a
	F_1	85	5.29 ± 0.09bA	83	5.64 ±1.36aB	84	5.88 ± 0.11aA	-	-
	F_2	93	5.28 ± 0.09bA	87	5.33 ± 0.07bC	82	5.89 ± 0.15aA	-	-
2nd instar	F_0	86	3.51 ± 0.08cA	82	4.44 ± 0.14abB	89	4.27 ± 0.12bB	11	5.73 ± 0.76a
	F_1	85	3.56 ± 0.07bA	83	4.83 ± 1.73aA	84	4.68 ± 0.13aA	-	-
	F_2	93	3.61 ± 0.07cA	84	4.68 ± 0.14aAB	78	4.00 ± 0.10bB	-	-
Female 3rd instar	F_0	47	3.72 ± 0.09bA	17	4.53 ± 0.21aA	45	4.36 ± 0.23aA	2	3.50 ± 0.50b
	F_1	45	3.29 ± 0.10bB	30	4.10 ± 0.15aA	40	4.78 ± 0.37aA	-	-
	F_2	49	3.53 ± 0.09bAB	33	4.03 ± 0.16aA	40	4.47 ± 0.24aA	-	-
Male 3rd instar	F_0	39	2.08 ± 0.15aA	65	2.45 ± 0.13aA	44	2.27 ± 0.17aA	9	2.44 ± 0.38a
	F_1	40	1.93 ± 0.11aA	53	2.11 ± 0.13aA	44	2.20 ± 0.15aA	-	-
	F_2	44	2.05 ± 0.13aA	51	2.41 ± 0.14aA	38	2.13 ± 0.17aA	-	-
Male pupa	F_0	39	2.44 ± 0.17bA	65	2.69 ± 0.16abA	44	2.50 ± 0.14bA	9	3.89 ± 0.59a
	F_1	40	2.52 ± 0.19aA	53	2.62 ± 0.16aA	44	2.20 ± 0.16aA	-	-
	F_2	44	2.50 ± 0.14bA	51	2.92 ± 0.14aA	38b	2.50 ± 0.16A	-	-

The data (Mean ± SE) followed by the same letters were not significantly different by paired bootstrap test ($P < 0.05$). Lowercase letters indicated comparison among different host plants, and capital letters indicated comparison among different generations.

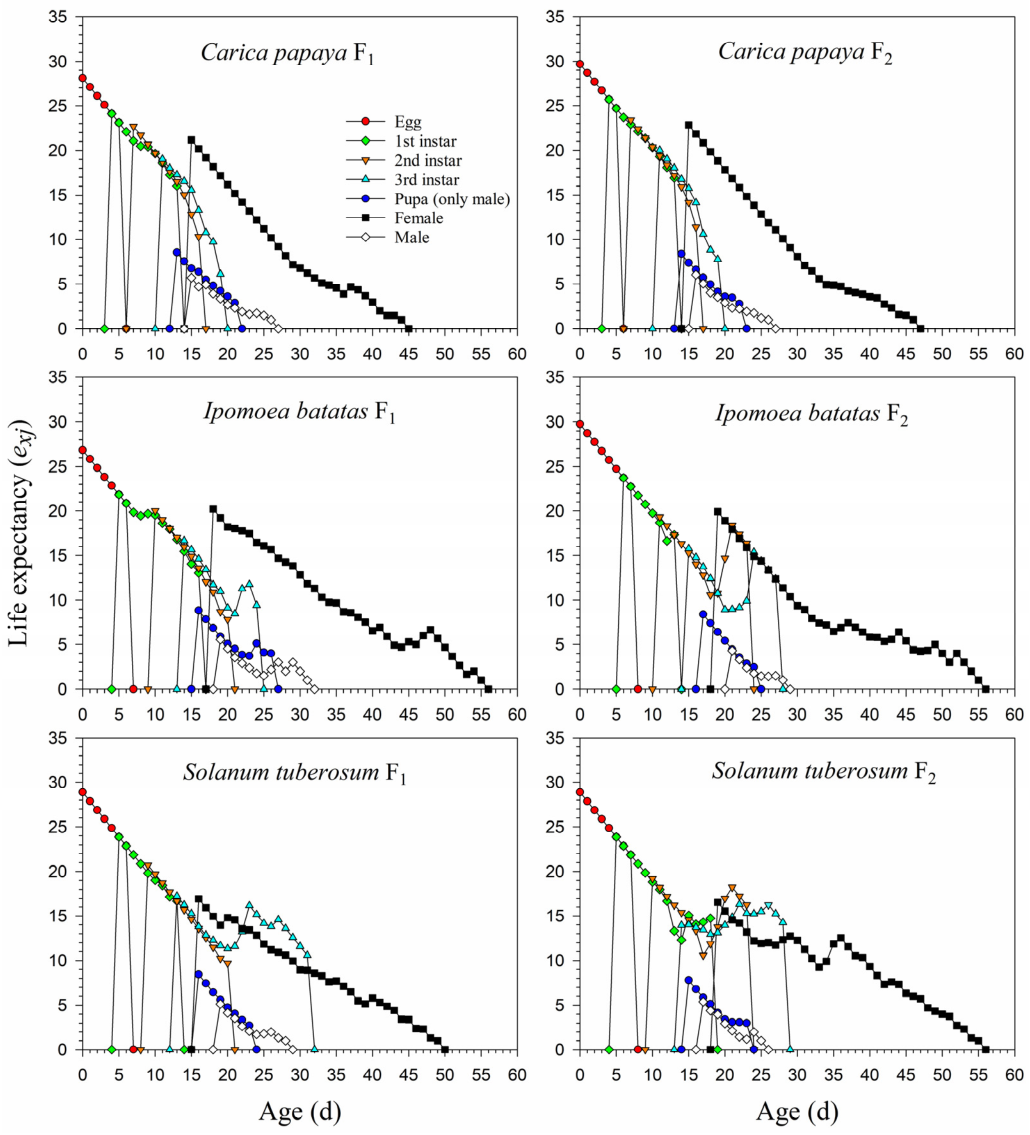

Supplementary Figure 1. Age-stage-specific life expectancy (e_{xj}) of *Paracoccus marginatus* reared on three different host plants (F_1-F_2).

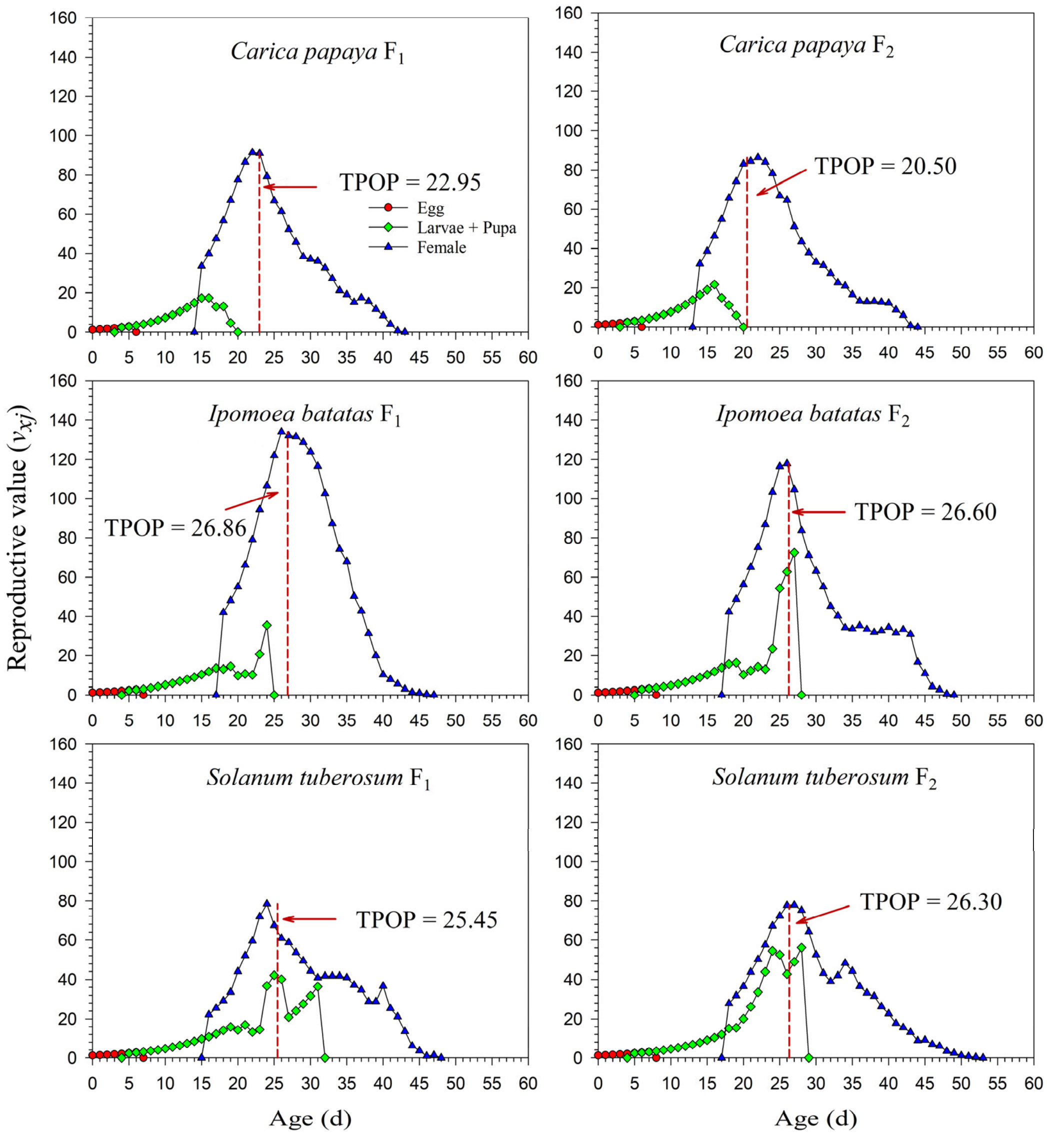

Supplementary Figure 2. Age-stage-specific reproductive value (v_{xj}) of *Paracoccus marginatus* reared on three different host plants (F_1-F_2). The red vertical dashed line in each figure denotes the total preoviposition period (TPOP).
